# Transforming healthcare: evaluating a decade of postgraduate training at the Liberia College of Physicians and Surgeons

**DOI:** 10.1186/s12960-026-01081-z

**Published:** 2026-05-11

**Authors:** Juul Marlies Bakker, Douglas Weru Nyaguthii, Benetta Collins Andrews, Philderald Pratt, John Mulbah, Alex J. van Duinen, Hakon Angell Bolkan

**Affiliations:** 1 Institute of Nursing and Public Health, Norwegian University of Science and Technology (NTNU), Postboks 8905, 7491 Trondheim, Norway; 2 CapaCare Liberia, Monrovia, Liberia; 3 Liberia College of Physicians and Surgeons (LCPS), Monrovia, Liberia; 4 Liberia Medical and Dental Council (LMDC), Monrovia, Liberia; 5 Department of Surgery, ELWA Hospital, Monrovia, Liberia; 6 Department of Surgery, St Olav’s Hospital, Trondheim University Hospital, Trondheim, Norway

**Keywords:** Medical education, Postgraduate training, Specialization, Health workforce, Human resources, Evaluation, Liberia, West Africa

## Abstract

**Background:**

Postgraduate training in Liberia was commenced in 2013 under the Liberia College of Physicians and Surgeons (LCPS). Since its inception, 125 medical doctors have specialized in seven disciplines: internal medicine, pediatrics, surgery, obstetrics and gynecology, family medicine, ophthalmology and psychiatry. This study evaluates the outcomes of a decade of postgraduate training in Liberia.

**Methods:**

An online questionnaire was distributed to all graduates, collecting data on demographics, work history, motivation, self-assessed competencies, and training experiences, including examinations, research and feedback. Descriptive analysis was used to calculate response frequency distributions for quantitative outcomes, and thematic analysis was used for open answer responses.

**Results:**

Ninety graduates (72.0%) responded to the questionnaire. All respondents worked in Liberia, with 56.3% in Montserrado County. Most (94.4%) were primarily employed in the public sector, whereas 21.1% held additional roles in the private sector or academia. After 3 years of membership-level postgraduate training to become a specialist, 30.0% pursued additional education, including 18.9% fellowship training. Overall, graduates expressed strong confidence in competencies gained during the postgraduate training, particularly medical knowledge, professionalism and teaching, with over 95% of graduates considering themselves competent in these areas. In contrast, only 44.3% felt competent in research. Nearly all graduates reported that the training supported their career growth and would recommend it to colleagues. Suggestions for improvements included increasing faculty numbers and diversity, enhancing training resources, and expanding research opportunities.

**Conclusions:**

The LCPS has contributed substantially to developing a competent specialist health workforce in Liberia. Graduates had a high retention rate and were primarily employed in clinical roles in the public sector. Challenges remain, such as too few specialists and limited healthcare coverage in rural areas. To train well-equipped specialists, it is crucial to strengthen faculty, institutional capacity and training resources, and to build research capacity. Sustained investment and continued collaborations between the government and partners are needed to implement strategies to maintain retention and improve equitable distribution of specialists and to support their career growth. These efforts will ensure the program’s impact to meet Liberia’s evolving healthcare demands effectively.

**Supplementary Information:**

The online version contains supplementary material available at 10.1186/s12960-026-01081-z.

## Background

The workforce is the cornerstone of any health system and is playing a vital role in improving health service coverage [1]. In 2016, the World Health Organization introduced the “Workforce 2030”, a global strategy on human resources for health [2]. The strategy emphasizes achieving effective health coverage through adequate availability, acceptability, accessibility, quality, and service utilization. To enhance coverage, it is essential to optimize the health workforce, prepare for future needs, build institutional capacity, and strengthen data on human resources for health [2].

### Health workforce in Liberia

Liberia is a low-income country in West Africa with 5.3 million inhabitants [3]. The civil war between 1989 and 2003 severely affected the health system. The education of health workers collapsed and many practicing health care workers left the country, reducing the number of employees in the public health sector by 60% [4]. While Liberia was still rebuilding the health system and workforce after the war, it was faced with the West-African Ebola Virus disease outbreak in 2014–2016. Ebola claimed the lives of 8% of all healthcare workers, and other healthcare workers left their jobs [5–7].

Post-Ebola, Liberia continued to have one of the lowest densities of healthcare workers globally, with 0.04 physicians per 1000 people [8], far from the World Health Organization (WHO) estimate for adequate coverage at a minimum 2.5 per 1000 population. Most health services were delivered by non-specialist medical doctors [9]. A survey among medical school graduates showed that less than half of them assessed themselves as fully competent [10]. The Liberia Health Workforce Plan 2015–2021 outlined strategies and priorities to (re)build the health workforce [11]. Priority areas of intervention included increasing the number of health workers, improving education quality, and diversifying skills. The strategy targeted five key cadres: community health workers, health managers, registered midwives, registered nurses, and physicians (both general and specialist). For physicians, the aim was to raise annual medical school enrollment from about 40 in 2015 to 125. Despite limited specialty training programs in Liberia, the development of national capacity to produce specialists for strengthening of the medical and clinical education systems was emphasized. The target for specialist training enrollment increased from 18 to 24 annually by 2021 [11].

### Medical education

The A.M. Dogliotti School of Medicine under the University of Liberia is the only medical school in Liberia. Recently, its curriculum for medical education was restructured to reduce the time to acquire an undergraduate medical degree (M.D.) and to increase attrition rates. In addition, new faculty were trained in basic sciences and teaching methods [10]. Previously, students required to acquire a B.Sc. (4 years) prior to enrollment in medical school (5 years). In 2021, the reformed 7-year, integrated, competency-based program for a M.D, was approved [10]. After graduation from medical school, a 1-year internship rotation is required to be licensed by the Liberia Medical and Dental Council (LMDC). Medical education is free, and licensed medical doctors are absorbed by the Ministry of Health. A year of rural service is required before medical doctors can apply for postgraduate residency training [12].

### Establishment of the Liberia College of Physicians and Surgeons (LCPS)

In 2012, the LCPS was established by an Act of Legislation to train and qualify specialists in Liberia [13]. Initially, postgraduate medical residency training was offered in general surgery, internal medicine, obstetrics and gynecology, and pediatrics up to membership level. The training structure and curricula were modelled after regional programs of the West-African College of Physicians (WACP) and the West-African College of Surgeons (WACS). Residency programs, primarily based on John F. Kennedy Medical Center in Monrovia, include 3 years of training with rotations at various departments at additional nationally and regionally accredited hospitals, leading to LCPS membership after a final examination [14]. This can be followed by advanced subspecialist-level fellowship training, which is currently only accredited for few sub-specializations in Liberia. The programs are nationally accredited by the LCPS and regionally by the WACP and WACS. Having regional collaboration with WACP and WACS enhances opportunities for graduates to register with regional colleges and pursue additional fellowship education [15, 16]. Postgraduate training began in September 2013 but was suspended for 10 months during the Ebola outbreak. The first Liberian-trained specialists were inducted as members into the college in 2017. In the following years, additional faculties were established, including family medicine (2017), ophthalmology (2019), psychiatry (2019), and community medicine (2023).

Specialists are clinically trained, but are also expected to be the future trainers and researchers [17]. Specialists have an essential role in strengthening of the health system and to ensure sustainability and self-reliance of the training program. Initially, the postgraduate training program was heavily dependent on external resources for funding as well as for faculty. Both Dahn et al. [18] and Sanoe et al. [19] emphasize the importance of building academic and institutional capacity and infrastructure to successfully educate health workers. Several partnerships with international institutions were formed [16, 19, 20]. The faculty employed under the Health Workforce Program funded by the World Bank during the initial 5 years of postgraduate training played an important role in advancing the faculty pipeline. This has developed a foundation for mentorship and education of the next generation of specialists in Liberia. Most of the international faculty deployed under the program were from West Africa, which not only ensured alignment of the training with the accreditation requirements and curricula of WACP and WACS, but also strengthened the collaboration in the region.

### Current specialist workforce in Liberia and future planning

There are no unified figures for the actual number of medical doctors in Liberia. The WHO National Health Workforce Accounts reported 954 medical doctors in 2022, including 840 general practitioners and 114 specialists [21]. According to the LMDC, the number of specialists had increased from 15 to 120 Liberian specialists between 2012 and 2019, as well as 248 non-Liberian specialists [13]. Between 2013 and 2023, 125 Liberian-trained specialists across seven specialties were inducted by the LCPS. In 2018, most surgical specialists worked in or around the capital [9].

### Aim of this study

The LCPS has increased the number of specialists in Liberia. However, the impact of the program in terms of retention, employment locations, roles, and relevance of the knowledge and skills acquired by graduates from the postgraduate training program remain unclear. This study aims to evaluate the outcomes of a decade of postgraduate training in Liberia by assessing graduate demographics, employment history, and the relevance and utilization of acquired knowledge and skills. The results can be used to inform program improvements and guide continuous professional development efforts.

## Methods

### Study design

In this retrospective, cross-sectional study, all graduates of the LCPS membership program were requested to participate in a survey to examine the effectiveness and appropriateness of postgraduate training in Liberia. Primary outcomes of interest included graduate retention rates, geographical distribution of graduates, their job function and sector, and a self-assessed level of confidence in medical competencies. Graduation and retention rates are important indicators of the impact that postgraduate training has on the health workforce and health system in Liberia.

### Setting and study population

Liberia is divided into 15 counties. Approximately one-third of the population lives in Montserrado County [3]. This study included all medical doctors who completed their postgraduate training under the LCPS from inception in 2013 to September 2023. Residents who were still in training at the time of the study, who had dropped out during training, or Liberian specialists who specialized abroad were not eligible for inclusion.

### Study tools

An online questionnaire (Additional file 1) retrieved demographic information, educational background, current and previous work situation, and a self-evaluation of medical competencies gained during the postgraduate training. The 15 question self-assessment was framed to evaluate seven core competencies as defined by the LCPS [14]. These competencies included patient care, medical knowledge, practice-based learning and improvement, interpersonal and communication skills, professionalism, systems-based practice, and teaching. Participants were asked to evaluate each competence on a 5-point Likert scale (strongly disagree, disagree, neutral, agree, strongly agree). In addition, questions were asked about several aspects of the training, such as feedback, examination and research during the postgraduate training, and a reflection on their motivation before and after the training.

### Data collection

Data collection took place between September and December 2023. The questionnaire was disseminated to all 125 graduates of LCPS postgraduate training through the online platform Nettskjema [22]. All graduates were contacted by phone call, email, WhatsApp or text message, or a combination. Each person was contacted at least three times and in different ways before being deemed a non-responder. To optimize the response rate, two presentations were held at the main teaching hospital in Liberia (John F. Kennedy Medical Centre) and at the annual scientific meeting of the LCPS to explain the purpose of the study. In addition, several larger hospitals across Liberia were visited to distribute information about the study.

### Data analysis and ethical considerations

Data were exported from Nettskjema to Microsoft Excel (Microsoft Corporation, USA) for processing and descriptive statistics. Personal identifiers were removed prior to analysis. Where applicable, answers to open questions were transformed into categorical variables. Analysis was performed for the complete data set and stratified by specialization. Aggregated scores were calculated for Likert-scale scores and results are presented in medians or percentages. Open-ended responses were analyzed by the principal investigator using thematic analysis. Responses were organized in relation to training and health system factors. For both domains, positive aspects, challenges, and suggestions were categorized inductively.

The study was approved by the Atlantic Center for Research and Evaluation Institutional Review Board (ACRE IRB) in Liberia on 28 August 2023 (Reference number 23-08-383).

## Results

### Response rate

**Fig. 1:**
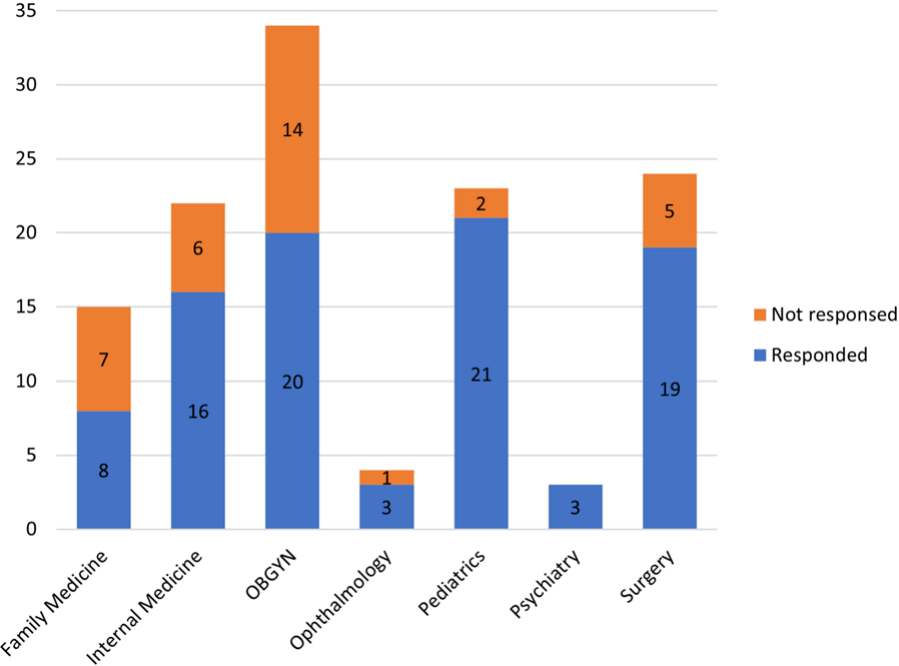
Total number of graduates and respondents by specialization

The response rate to the questionnaire was 72.0% (90 of 125) varying between 53.3% for family medicine and 100.0% for psychiatry. For all specialists’ tracks, the response rate was more than 50% (Fig. 1). The response rate by year of specialization ranged from 43.8 (2019) to 87.5% (2018). For the majority of the 34 non-responders, the reason not to participate is unknown, whereas two could not complete the questionnaire due to internet problems, and one lacked time to participate.

### Demographics

Sixty percent of the respondents was male and 40.0% female, compared to 64.0 and 36.0% of all graduates, respectively (Table 1). The median age at graduation was 37.5 years (IQR 34–42 years). Most respondents (92.2%) were Liberian nationals. Twenty-seven (30.0%) had pursued an additional education after completing the membership level of postgraduate training. The most common additional training were postgraduate fellowship training (17 of 90), or a master’s degree (7 of 90), most often within public health. The majority (76.6%) finalized their training within 3 years. Two-thirds of those who took 4–5 years were in the first cohort that started in 2013, whose training was affected by the Ebola outbreak.

**Table 1:** Demographics, employment and education status of respondents

		Graduates (*n* = 125)	Respondents (*n* = 90)
Gender	Female	45 (36.0%)	36 (40.0%)
	Male	80 (64.0%)	54 (60.0%)
Median years between medical school and residency training (IQR)		–	2 (2–4)
Median age at end of residency program (IQR)		–	37.5 (34–42) years
Nationality	Liberian	–	83 (92.2%)
	Other	–	7 (7.8%)
Current function	Clinical	94 (75.2%)	70 (77.8%)
	Specialist	–	51 (56.7%)
	Consultant	–	17 (18.9%)
	Fellow	–	2 (2.2%)
	Clinical/admin	8 (6.4%)	13 (14.4%)
	Medical director	–	13 (14.4%)
	Administrative	11 (8.8%)	6 (6.7%)
	MOH/CHO^#^	–	3 (3.3%)
	Administrative	–	3 (3.3%)
	Other	12 (9.6%)	1 (1.1%)
	Leave	7 (5.6%)	–
	Other	5 (4.0%)	1 (1.1%)
Position	Full-time	–	89 (98.9%)
	Part-time	–	1 (1.1%)
Sector	Public	102 (81.6%)	85 (94.4%)
	Private	9 (7.2%)	3 (3.3%)
	Other	14 (11.2%)*	2 (2.2%)
Work location	Urban (Montserrado)	64 (51.2%)	49 (54.4%)
	Rural (other counties)	49 (39.2%)	38 (42.2%)
	Abroad/other	12 (9.6%)	3 (3.3%)
Additional work	Yes	–	19 (21.1%)
	Private practice	–	10 (11.1%)
	Teaching	–	6 (6.7%)
	Other	–	3 (3.3%)
	No	–	71 (78.9%)
Median months working in current job (IQR)		–	24 (11–52)
Duration of residency training	Within 3 years	–	69 (76.7%)
	More than 3 years	–	21 (23.3%)
Additional education	Master’s degree	–	7 (7.8%)
	Fellowship	–	17 (18.9%)
	Other	–	3 (3.3%)
	None	–	63 (70%)
Co-authored publications	None	–	37 (41.1%)
	1–2	–	32 (35.6%)
	3–5	–	12 (13.3%)
	> 5	–	9 (10.0%)

^#^MOH, Ministry of Health; CHO, County Health Office

*Includes 7 on leave (study/absence)

### Work location and history

Most of the graduates (94.4%) worked in the governmental sector, whereas 3.3% worked in the private sector and 2.2% in other sectors (Table 1). Even though only one person specified to work in a part-time position, 21.1% of the respondents indicated to have a second job next to their first employment. These second employments were mostly in the private sector or in teaching and took place in evening hours or during weekends.

**Fig. 2:**
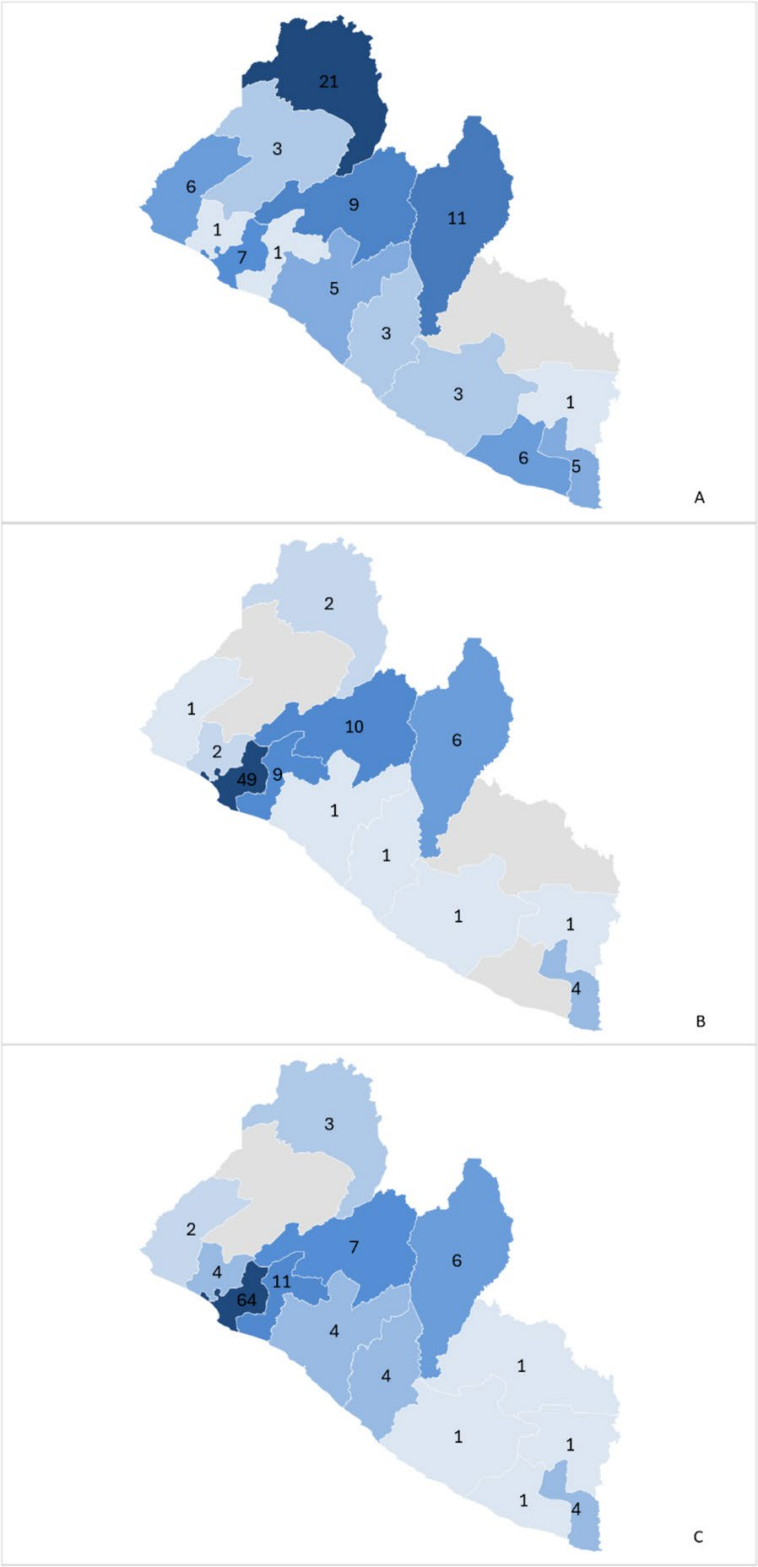
County of origin of respondents (**A**, *n* = 83), work location of respondents (**B**, *n* = 87), and work location of all graduates (*n* = 113)

Respondents originated from all but one county in Liberia (Fig. 2a). According to the LCPS, 12 graduates were currently abroad (9.6%). This study found that after residency training, 54.4% of the respondents worked in Montserrado County, where 36.7% of the population lived. In eight counties there was only one or no respondent (Fig. 2b, c). This was similar to the distribution among all graduates, where 51.2% worked in Montserrado. The median time respondents worked in their current job was 24 months (IQR 11–52). Forty-three respondents (47.8%) provided information about their previous work history. Out of these 43, eleven (25.6%) remained in the same county. Out of the 23 people who worked in Montserrado prior to their current job, only eight remained in Montserrado, whereas 15 are currently working in other counties. Specialists in Obstetrics and Gynecology were most spread with graduates working in nine different counties, and 47.9% working outside Montserrado County.

### Evaluation of competence

**Fig. 3:**
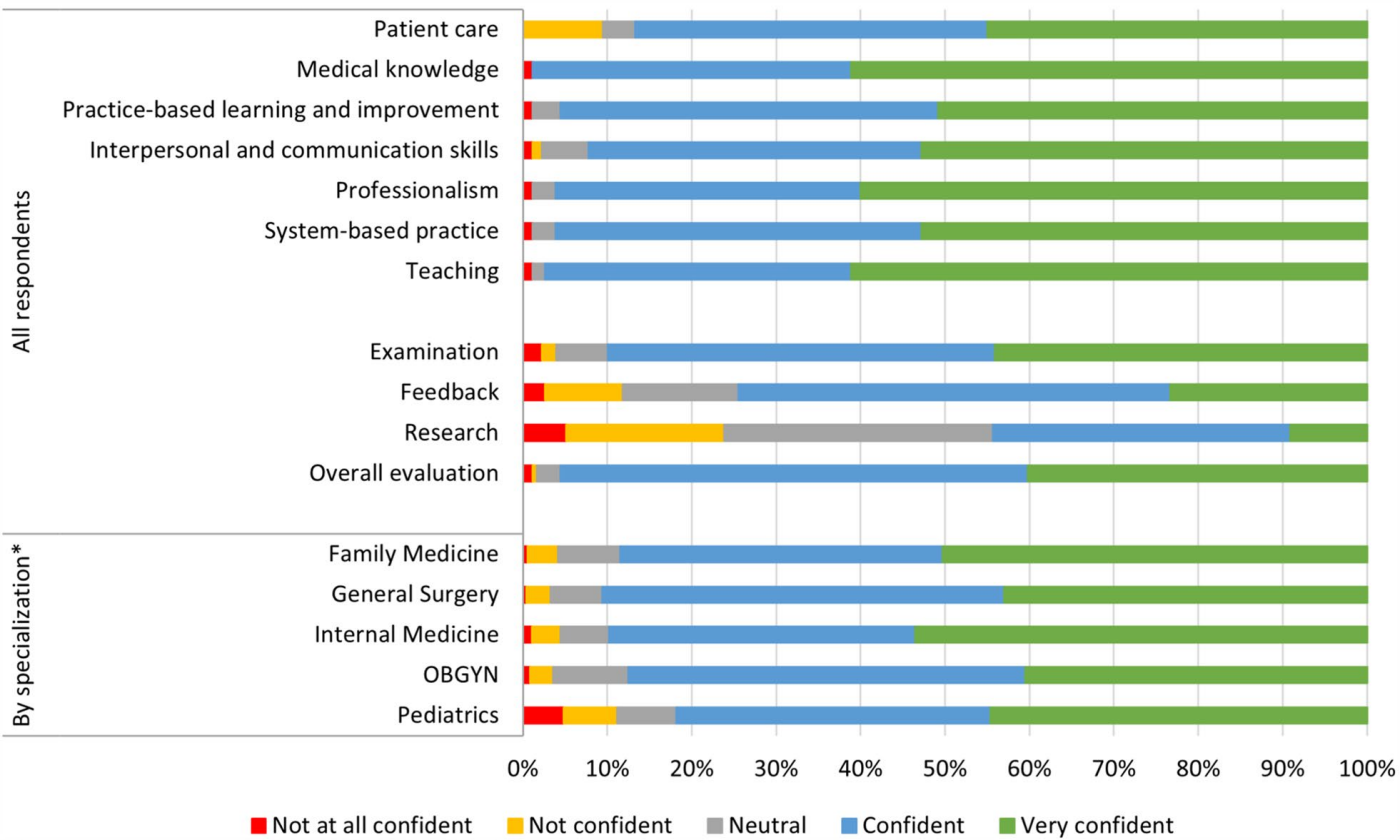
Overview of responses by competencies and by specialization. *Excluding ophthalmology (*n* = 3) and psychiatry (*n* = 3)

Overall, the graduates were confident with the competencies gained during the postgraduate training (Additional file 1: Table S1). The highest rated competencies were medical knowledge, teaching, professionalism and system-based practice with over 96% of graduates assessing themselves as competent. Whereas 95.5% were confident in integrating scientific evidence into their clinical decision-making, only 44.3% felt competent to perform research. 74.4% indicated that the curriculum adequately covered all clinical subjects that they need in their work (Fig. 3). Although 50.6% of the respondents agreed with the statement that they had opportunities to engage in research activities, 58.9% of the respondents had (co-)authored research publications at the time of study. Almost all graduates (95.5%) reported that the training has provided them with opportunities for career growth and would recommend it to junior colleagues. There were only slight differences between the various specializations. Graduates in psychiatry rated their competences highest with an average score of 88.3% of the maximum, while this was lowest for pediatrics with 81.4%.

### Motivational factors

**Fig. 4:**
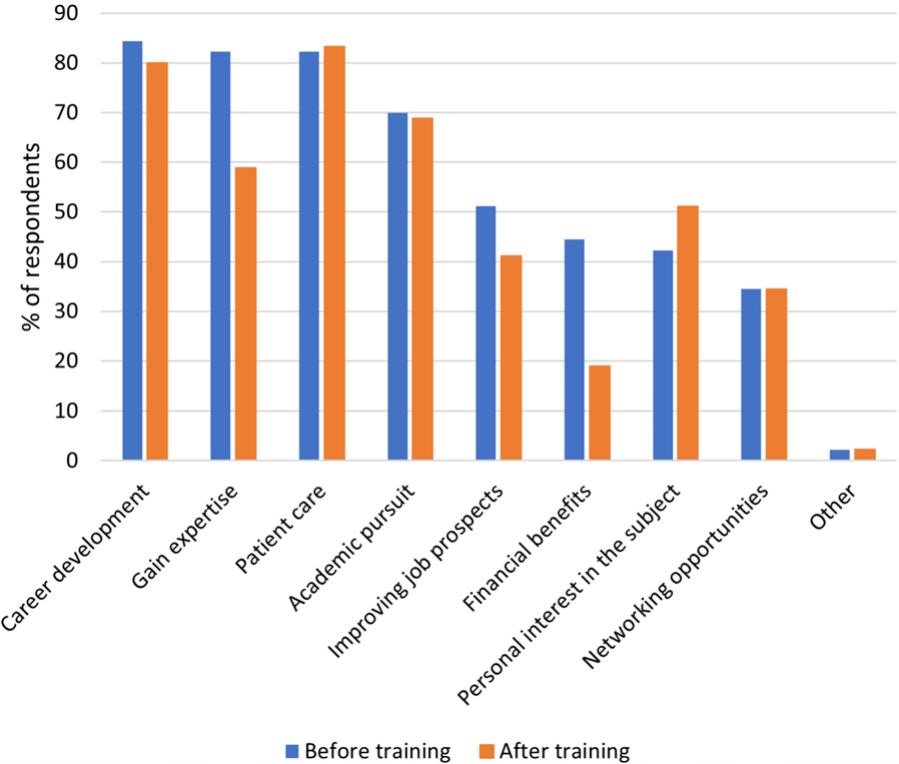
Motivational factors before and after postgraduate training

The main motivational factors to enroll in postgraduate training were interest in patient care, personal interest in the subject, and a desire to become an expert in the field. Motivational factors that decreased between enrollment and graduation of postgraduate training were financial benefits (44.8–18.4%) and desire to become expert in the field (83.9–58.6%) (Fig. 4). External factors, such as financial benefits, career development, and improving job prospects reduced more than patient-related factors or clinical aspects.

### Graduates’ suggestions

Thirty-eight graduates offered open-ended replies and expressed both strengths and challenges with the training program, suggestions for improvement of the program, and effect on the health system. Several graduates praised the program for enhancing their medical knowledge and skills, enabling them to deliver high-quality patient care. Common criticisms on the training included the lack of adequate mentorship due to overburdened faculty, insufficient training resources, such as laboratory and imaging facilities, and poor infrastructure, including inadequate resident housing. Suggestions for improvement of the training program included increasing faculty numbers, especially in subspecialties, improving research and publishing training, providing better investigative tools, and enhancing collaboration with external organizations and faculty during rotations. Graduates emphasized the importance of postgraduate training in improving the health system and contributing to the national health workforce. Addressing salary disparities and career progression post-graduation were highlighted as key areas for improvement.

## Discussion

### Impact of postgraduate training in Liberia on the health system

This study examines 10 years of postgraduate training in Liberia, highlighting a high domestic retention rate among graduates, most of whom contribute to clinical service delivery in the public sector. The competencies gained through the postgraduate training were perceived relevant and well-aligned with their current work responsibilities.

Before the establishment of postgraduate training, Liberia relied on regional and international support to train its doctors, impeding affordability, accessibility, and retention [13]. Many trained abroad did not return, exacerbating the scarcity of specialists within the country [23, 24]. The establishment of postgraduate training in Liberia addressed these challenges by building local capacity to produce specialists tailored to the nation’s specific health system needs. The local training of 125 specialists represents a significant milestone, directly contributing to improving access to specialized healthcare services and reducing the dependency on foreign-trained professionals. To become self-reliant, the LCPS Faculty Sustainability Plan in 2022 expected to need at least five more years of visiting and sub-specialist faculty, as well as a progressive increase of the government budget allocation toward the training [17]. Until then, there is still an important role for collaboration.

Postgraduate training is mostly hands-on, in-service training, and thus the quality and level of service delivery in the system, where people are trained directly influences the quality of the training. Therefore, training programs can boost service delivery and vice versa. The graduated specialists have strengthened the public health facilities and likely enhanced clinical service delivery. The lowest ranked medical competence was patient care. Better diagnostic possibilities and more (subspecialist) faculty could improve this. Looking ahead, greater emphasis should also be placed on strengthening academic competencies, especially since the LCPS training programs also aim to strengthen academic and teaching capacity. Research was identified by graduates as their least developed skill. Understanding and practicing research during residency have been associated with better clinical performance and are crucial for practicing context adapted evidence-based medicine [25, 26].

### Retention and distribution

A significant challenge in healthcare systems in low-income countries is attracting and retaining health workers in the country after training, and to distribute them according to the needs [27]. The “brain drain” resulting from migration of well-trained health workers is a major issue in some West-African countries, with Nigeria and Ghana being among the largest “supplier countries” [24]. Among the specialists in Liberia, there was mostly temporary migration for education purposes. A study among surgical specialists trained under the Colleges of Surgeons of East, Central and Southern Africa (COSECSA) also found high retention rates with 85% of graduates remaining in the country, where they were trained [28]. The postgraduate training is relatively young, and long-term retention could, therefore, not be assessed.

For Liberian candidates, there are no tuition fees for the postgraduate training, and specialists are absorbed by the public health system after graduation. In this study, internal migration from the public to the private sector was low. However, in recent years, there has been a rapid increase in private health facilities in Liberia, particularly in urban areas [29]. This might cause a shift in the future that can potentially pose a challenge to equitable access to healthcare services.

We observed that more than half of the graduates worked in Montserrado county, while in rural areas the health care system still heavily relies on non-specialist medical doctors and non-physicians to provide care [9]. The unequal distribution of specialists will hamper the goal of achieving universal health coverage [30]. Decisions to relocate to, stay in, or move from a rural area are influenced by several factors, such as financial aspects, working and living conditions, family and community, job and education opportunities, and mandatory services or placement [27, 31]. The concentration of training institutions, such as the medical school and the main teaching hospital, in Monrovia, contributes to this urban–rural divide. Joharifard et al. described a significant change in surgical procedures in a rural hospital in Liberia after the arrival of a surgeon, with increased variety and complexity [32]. Apart from increasing access to quality services, employing specialists in rural regions also has the potential to build the capacity of health facilities and to transform regional or district hospitals into more attractive training sites (e.g., for interns/medical doctors, and nursing students) [19]. Training health workers locally has the added advantage of higher retention rate, in addition to better preparing them for the health system and context, where they will work [27, 33].

### Implications for the future: health workforce strategies

Despite progress in addressing the health worker crisis in Liberia over the last decade, an important gap remains between the current specialist health workforce and the need for health services. With an expected population growth and current production rate of specialists, this gap is expected to increase further [3]. Several strategies have demonstrated the potential to effectively address health labor market challenges. These can broadly be divided into three main categories: strategies focusing on the production of health workers (education), addressing the inflows and outflows of qualified health workers (such as migration, retention, and recruitment of unemployed health professionals), and policies aiming to address the maldistribution of the workforce and inefficiencies. The latter includes strategies to improve the productivity and performance of the existing health workforce, resolve skills–mix disparities, and promote the redistribution and retention of health workers in underserved regions [2, 34].

To mitigate the clustering of specialists in certain regions in Liberia, it is crucial to tackle structural causes. In the near future, efforts could focus on recruiting students from rural areas and integrating rural exposure into training, while in the long term educational institutions could be established in different regions. Liberia’s public sector retains most specialists post-training, offering the government an opportunity to influence workforce distribution. However, producing sufficient specialists remains a challenge, with only 15 graduates annually and the additional burden of fulfilling roles in service delivery, administration and governance, academia, and education. Mandatory rural service, expanding rural workers' scope, and creating specialized workforce categories can help address these gaps [27]. Policies must balance strengthening primary care systems, which are essential for cost-effective, equitable health coverage, with increasing access to specialist care. Specialist training should align with broader health system goals, balancing the workforce mix to enhance efficiency and sustainability [35]. Task-sharing has been an integrated part of the Liberian health system since the 1960s through its physician assistant training program [36], and the more recently implemented task-sharing program for experienced midwives to perform emergency obstetric and neonatal care [37]. While expanding task-sharing could alleviate Liberia’s health workforce shortage in the near term until there are more specialists, conflicting views among medical doctors and stakeholders constrain its scale-up into different areas [38].

Establishing structures for financial advancement, incentives, and continuous professional development are essential to maintain a skilled, productive and motivated workforce. These are even more important in underserved regions to advance equitable universal health coverage and ensuring access to specialized care nationwide [39]. The narratives of specialists working in rural areas emphasized the lack of resources and diagnostic tools to work with, resulting in frustration and demotivation. These challenges ultimately push professionals to return to urban areas or to transition to private or dual practice. Addressing health worker factors (e.g., knowledge, skills, and motivation), work factors (e.g., clinical guidelines), and the health facility environment (e.g., caseload, supervision, and availability of equipment and supplies) not only supports retention but also ensures the sustained high-quality performance of health workers [40]. Further research is needed to clarify what the key factors are in the Liberian context, and to examine the determinants of retention and migration among Liberian specialists.

### Strengths and limitations

This study provides a comprehensive overview of the impact of the first 10 year postgraduate training in Liberia. The response rate among the graduates contributes to the legitimacy of the study, although we cannot account for 28% of graduates. Respondents included graduates from all regions and specialties. However, rural areas might be under-represented due to challenges in participating in an online questionnaire. The retrospective, survey-based methodology introduces a recall bias. The relatively small number of graduates limited the possibility of subgroup analysis. Graduates performed a self-evaluation of the competences gained during postgraduate training, which provides a subjective view. Answers were not validated with their job tasks and the services they delivered. Accuracy of self-assessment is limited, and the results might, therefore, not correlate with their actual performance [41]. In addition, graduates are mostly posted by the Ministry of Health to certain positions, and do not select their work location, which might influence the appropriateness and use of certain competences and could influence their evaluation of the training. The impact of the postgraduate training program in terms of clinical outcomes cannot be assessed based on these findings. Finally, this study focused on graduates from the LCPS training, and results are, therefore, not representative of the entire specialist workforce.

## Conclusion and recommendations

The LCPS has made significant strides in building the country’s specialist health workforce over the past decade, but challenges remain. Addressing faculty shortages, building institutional and academic capacity, improving training resources, and promoting research are critical to ensuring that the program produces well-rounded specialists. Furthermore, tackling geographic disparities in the distribution of specialists, enhancing career progression pathways, and fostering external collaborations will strengthen the LCPS program and improve healthcare outcomes in Liberia. Continuous investment in postgraduate training and professional development is essential to meet the evolving healthcare needs of Liberia and build a resilient and skilled specialist health workforce.

## Supplementary Information

**Additional file 1.**

**Additional file 2.**

## Data Availability

The datasets generated and analyzed during the current study are available from the corresponding author on reasonable request.

## Abbreviations

ACRE IRB: Atlantic Center for Research and Evaluation Institutional Review Board
LCPS: Liberia College of Physicians and Surgeons
LMDC: Liberia Medical and Dental Council
WACP: West-African College of Physicians
WACS: West-African College of Surgeons
WHO: World Health Organization
