# Supplementary material for: Transforming healthcare: evaluating a decade of postgraduate training at the Liberia College of Physicians and Surgeons
Source: Hum Resour Health. 2026 May 11;24:35. doi: 10.1186/s12960-026-01081-z (PMC13330417; doi:10.1186/s12960-026-01081-z)
Supplement: Supplementary file 1 — Additional file 1. [file 12960_2026_1081_MOESM1_ESM.docx]

**Additional file 1 – Questionnaire**

**Personal information**

Name:

Email address:

1. Gender:  Male  Female  Other/ Wish not to disclose.
2. Age: _________ (years)
3. Nationality:  Liberian  Other , If other, which: ________________
4. County of origin: ________________

**Education history**

1. Specialization:

 Internal Medicine  OB/GYN  Pediatrics  General Surgery  Family Medicine

Ophthalmology  Psychiatry

1. Year of graduation from medical school: _____________ (year)
2. Year of starting postgraduate specialization training:______________
3. Year of graduation from postgraduate specialization training:______________
4. Additional education after postgraduate membership training

(e.g. master’s degree, PhD, fellowship):  Yes  No

If yes, please specify which education: __________________________________, Duration of additional education (in months)______________________________

**Employment after graduation**

***Current job***

1. Job function: ________________________________

Is your current job position full-time?  Yes  No

Type of employment:  Governmental  Private sector

 Non-profit sector  Other  Not employed

If ‘other’ or ‘not employed’, please specify____________________________________

Current workplace (county): _______________________________________________ Time you started this employment (month/year):________________________________

1. Do you currently have another job next to your main employment (e.g. private practice, volunteering, etc.)?  Yes  No
2. Please specify your secondary employment (if applicable): _______________________
3. How is your time divided between the different jobs (if applicable)?_________________

***Previous employment***

**We would like to know your employment history since you finished your postgraduate training. Please enter the information for the last 3 jobs you had, if applicable.**

1. Previous employment (up to 3 previous employments):
   1. Job function: ________________________________

Type of employment:  Governmental  Private sector

 Non-profit sector  Other  Not employed

Work location (county): _____________________________________________ Time you started this employment (month/year):__________________________

- 1. Job function: ________________________________

Type of employment:  Governmental  Private sector

 Non-profit sector  Other  Not employed

Work location (county): _____________________________________________ Time you started this employment (month/year):__________________________

- 1. Job function: ________________________________

Type of employment:  Governmental  Private sector

 Non-profit sector  Other  Not employed

Work location (county): _____________________________________________ Time you started this employment (month/year):__________________________

**Competencies**

We will ask questions about the competencies you have gained during your postgraduate training, related to different aspects of your work, such as: patient care, medical knowledge, practice-based learning, communication, professionalism, teaching, etc.

Please indicate to which degree you agree with the following statements.

*Patient care*

1. During my postgraduate training the curriculum adequately covered all clinical subjects that I need in my current job.

Strongly disagree  Disagree  Neutral  Agree  Strongly agree  No answer

1. I learned how to identify and manage common / relevant medical conditions and emergencies

Strongly disagree  Disagree  Neutral  Agree  Strongly agree  No answer

*Medical knowledge*

1. The medical knowledge I gained during my training program helped me to develop critical thinking and problem-solving skills necessary for effective patient care.

Strongly disagree  Disagree  Neutral  Agree  Strongly agree  No answer

1. The LCPS training has improved my clinical skills and knowledge.

Strongly disagree  Disagree  Neutral  Agree  Strongly agree  No answer

*Practice based learning and improvement*

1. I feel confident to implement quality improvement methods in my daily practice after completing the residency program.

Strongly disagree  Disagree  Neutral  Agree  Strongly agree  No answer

1. I can find, assess, and integrate scientific evidence into my clinical decision-making process.

Strongly disagree  Disagree  Neutral  Agree  Strongly agree  No answer

*Interpersonal and communication skills*

1. During the training there was sufficient attention for communication skills when interacting with patients and their families.

Strongly disagree  Disagree  Neutral  Agree  Strongly agree  No answer

1. The training program has enhanced my ability to work effectively within a multidisciplinary team.

Strongly disagree  Disagree  Neutral  Agree  Strongly agree  No answer

*Professionalism*

1. The training has taught me to interact with diverse patient populations while maintaining patient privacy and autonomy.

Strongly disagree  Disagree  Neutral  Agree  Strongly agree  No answer

1. The training emphasized the importance of medical record-keeping.

Strongly disagree  Disagree  Neutral  Agree  Strongly agree  No answer

*System-based practice*

1. My training has prepared me to improve the healthcare system I am currently working in.

Strongly disagree  Disagree  Neutral  Agree  Strongly agree  No answer

1. I can implement potential solutions to contribute to improving patient care systems.

Strongly disagree  Disagree  Neutral  Agree  Strongly agree  No answer

*Teaching*

1. The teaching during my postgraduate training was engaging and interactive.

Strongly disagree  Disagree  Neutral  Agree  Strongly agree  No answer

1. I feel confident in my ability to educate patients and families about their condition and treatment.

Strongly disagree  Disagree  Neutral  Agree  Strongly agree  No answer

1. I feel confident in my ability to educate students and other health professionals about best practices and evidence-based care.

Strongly disagree  Disagree  Neutral  Agree  Strongly agree  No answer

**Training components**

In the following questions we will ask you to evaluate several aspects of the postgraduate training program, such as examination, feedback, and research.

*Examination*

1. The examination was conducted in a fair and unbiased manner.

Strongly disagree  Disagree  Neutral  Agree  Strongly agree  No answer

1. The examination effectively evaluated my knowledge and skills as a medical professional.

Strongly disagree  Disagree  Neutral  Agree  Strongly agree  No answer

*Feedback*

1. The feedback I received during the postgraduate training was detailed and thorough.

Strongly disagree  Disagree  Neutral  Agree  Strongly agree  No answer

1. During the training, I regularly got feedback that helped me identify areas where I needed to improve.

Strongly disagree  Disagree  Neutral  Agree  Strongly agree  No answer

1. I felt the faculty was attentive to my training needs.

Strongly disagree  Disagree  Neutral  Agree  Strongly agree  No answer

*Research*

1. The training I received prepared me to conduct research independently.

Strongly disagree  Disagree  Neutral  Agree  Strongly agree  No answer

1. I had opportunities to engage in research activities.

Strongly disagree  Disagree  Neutral  Agree  Strongly agree  No answer

1. Number of (co-)authored papers: _____

*Overall evaluation of training*

1. The training has provided me with opportunities for career growth.

Strongly disagree  Disagree  Neutral  Agree  Strongly agree  No answer

1. I would recommend the training to colleagues.

Strongly disagree  Disagree  Neutral  Agree  Strongly agree  No answer

1. Why would you (not) recommend the postgraduate training program:
2. Which factors motivated you to specialize and to enroll in the postgraduate training in Liberia? (Select all that apply)

Financial benefits  Networking opportunities Improving my job prospects

 Career development  Interest in patient care  Personal interest in the subject

 Academic pursuit Desire to become an expert in the field

Other (please specify) _______________________________________________

1. In retrospect, in which areas were your expectations met? (Select all that apply)

Financial benefits  Networking opportunities Improving my job prospects

 Career development  Interest in patient care  Personal interest in the subject

 Academic pursuit Desire to become an expert in the field

Other (please specify) _______________________________________________

Final comments or suggestions:
