# Supplementary material for: Transforming healthcare: evaluating a decade of postgraduate training at the Liberia College of Physicians and Surgeons
Source: Hum Resour Health. 2026 May 11;24:35. doi: 10.1186/s12960-026-01081-z (PMC13330417; doi:10.1186/s12960-026-01081-z)
Supplement: Supplementary file 2 — Additional file 2. [file 12960_2026_1081_MOESM2_ESM.docx]

**Supplementary Table 1: Overview of responses to Likert-scale questions about postgraduate training**

| Competence | Question | Strongly disagree | Disagree | Neutral | Agree | Strongly agree | | Total |
| --- | --- | --- | --- | --- | --- | --- | --- | --- |
|  |  | **Number of respondents** | | | | | | |
| Patient care | During my postgraduate training the curriculum adequately covered all clinical subjects that I need in my current job | 0 | 16 | 7 | 47 | 20 | 90 | |
|  | I learned how to identify and manage common / relevant medical conditions and emergencies | 0 | 1 | 0 | 28 | 61 | 90 | |
| Medical knowledge | The medical knowledge I gained during my training program helped me to develop critical thinking and problem-solving skills necessary for effective patient care. | 1 | 0 | 0 | 36 | 53 | 90 | |
|  | The LCPS training has improved my clinical skills and knowledge | 1 | 0 | 0 | 32 | 57 | 90 | |
| Practice-based learning and improvement | I feel confident to implement quality improvement methods in my daily practice after completing the residency program. | 1 | 0 | 3 | 36 | 50 | 90 | |
|  | I can find, assess, and integrate scientific evidence into my clinical decision-making process. | 1 | 0 | 3 | 44 | 41 | 89 | |
| Interpersonal and communication skills | During the training there was sufficient attention for communication skills when interacting with patients and their families. | 1 | 2 | 8 | 35 | 44 | 90 | |
|  | The training program has enhanced my ability to work effectively within a multidisciplinary team. | 1 | 0 | 2 | 36 | 51 | 90 | |
| Professionalism | The training has taught me to interact with diverse patient populations while maintaining patient privacy and autonomy | 1 | 0 | 3 | 27 | 59 | 90 | |
|  | The training emphasized the importance of medical record-keeping. | 1 | 0 | 2 | 38 | 49 | 90 | |
| System-based practice | My training has prepared me to improve the healthcare system I am currently working in | 1 | 0 | 4 | 37 | 48 | 90 | |
|  | I can implement potential solutions to contribute to improving patient care systems | 1 | 0 | 1 | 41 | 47 | 90 | |
| Teaching | The teaching during my postgraduate training was engaging and interactive | 1 | 0 | 4 | 46 | 39 | 90 | |
|  | I feel confident in my ability to educate patients and families about their condition and treatment. | 1 | 0 | 0 | 23 | 66 | 90 | |
|  | I feel confident in my ability to educate students and other health professionals about best practices and evidence-based care. | 1 | 0 | 0 | 29 | 60 | 90 | |
| Examination | The examination was conducted in a fair and unbiased manner. | 2 | 1 | 8 | 37 | 41 | 89 | |
|  | The examination effectively evaluated my knowledge and skills as a medical professional. | 2 | 2 | 3 | 45 | 38 | 90 | |
| Feedback | The feedback I received during the postgraduate training was detailed and thorough | 3 | 9 | 11 | 47 | 20 | 90 | |
|  | During the training, I regularly got feedback that helped me identify areas where I needed to improve. | 1 | 12 | 10 | 43 | 24 | 90 | |
|  | I felt the faculty was attentive to my training needs | 3 | 4 | 16 | 48 | 19 | 90 | |
| Research | The training I received prepared me to conduct research independently | 5 | 19 | 31 | 27 | 7 | 89 | |
|  | I had opportunities to engage in research activities. | 4 | 14 | 25 | 35 | 9 | 87 | |
| Overall evaluation | The training has provided me with opportunities for career growth. | 0 | 1 | 3 | 49 | 37 | 90 | |
|  | I would recommend the training to colleagues. | 2 | 0 | 2 | 50 | 35 | 89 | |
